# Therapeutic Notes

**Published:** 1897-01-09

**Authors:** 


### THERAPEUTIC NOTES.

### LORETIN.

Loretin, one of the many proposed substitutes for iodoform, has a chemical descriptive title of meta-iodo-ortho-oxy-chinolin-ana-sulphonic acid. It is a yellow, odourless, crystalline powder, very slightly soluble in water and alcohol, but forming soluble salts with alkalies except with lime; it is non-poisonous and non-irritating. It possesses remarkable antiseptic properties, and exercises a gentle and uniform stimulative action or granulations. Its antiseptic properties are probably due to the liberation of iodine by the vital activity of the living tissues with which it comes in contact. The various forms in which Loretin may be applied are not generally known, and consequently we give a few of the most useful formulæ in which it may be ordered by the surgeon. For urethral bougies it may be combined with cocoa butter and employed in place of Mr. Watson Cheyne's iodoform bougies. Loretin, 5 per cent.; cocoa butter, 95 per cent. For incipient boils a useful plaster may be made in combination with mercury in the proportions of 2 per cent. and 6 per cent. respectively, made up with some guttapercha basis; or it may be applied in the form of collodion and painted on the surface. Loretin, 5 per cent.; alcohol, 10 per cent.; elastic collodion, 85 per cent. Or, better still, it may be combined with Filmogen (Schiff) prepared by Thomas Christy and Co., a new and excellent form of varnish. Loretin gauze may be prepared in the following manner. Absorbent gauze of the best quality should be drawn twice through a solution containing 6 per cent. Loretin, 1 per cent. calcined carbonate of soda, and 93 per cent. distilled water; the gauze should then be wrung out and dried. Many other forms of powder, suppositories, and solutions may be employed, for the formulæ of which the reader is referred to a special leaflet by Eugen Dieterich.

